# News from the editors of *Fluids and Barriers of the CNS*

**DOI:** 10.1186/2045-8118-11-13

**Published:** 2014-06-17

**Authors:** Lester R Drewes, Hazel C Jones, Richard F Keep

**Affiliations:** 1 Department of Biochemistry and Molecular Biology, University of Minnesota Duluth Medical School, Duluth, MN 55812, USA; 2 Gagle Brook House, Chesterton, Bicester OX26 1UF, UK; 3 Department of Neurosurgery, University of Michigan, Ann Arbor, MI 48105, USA

## Abstract

This editorial announces a new affiliation between *Fluids and Barriers of the CNS (FBCNS)* and the *International Brain Barriers Society (IBBS)* with mutual benefits to the journal and to society members*.* This is a natural progression from the appointment of two new Co-Editors in Chief: Professor Lester Drewes and Professor Richard Keep in 2013. FBCNS provides a unique and specialist platform for the publication of research in the expanding fields of brain barriers and brain fluid systems in both health and disease.

## Editorial

### Affiliation with the international brain barriers society

We are very pleased to announce that *Fluids and Barriers of the CNS (FBCNS)* is now affiliated to the *International Brain Barriers Society (IBBS). IBBS* is an international scientific and educational organisation that encourages and advocates basic and clinical research on the biological barriers in the central nervous system. Furthermore, *IBBS* promotes the dissemination of new knowledge about CNS fluids and barriers to scientists, physicians, patients, policy makers, public and private funding agencies and other concerned parties. The international society was founded in 2008 and is led by an international steering council headed by Professor Lester Drewes, University of Minnesota. The Society is an advocate for research into the neurovascular unit/blood–brain barrier and blood-cerebrospinal fluid barrier (NVU/BBB and BCSFB) and associated fluids. It promotes dialogue and exchange between scientists through affiliated conferences such as Cerebrovascular Biology, Blood–brain Barrier Consortium (Oregon), Gordon Research (Barriers of the CNS), Signal Transduction in the Blood–brain Barriers and others. It also awards bursaries to young scientists enabling them to attend conferences.

### Benefits of this affiliation

*Fluids and Barriers of the CNS* originally started as *Cerebrospinal Fluid Research* and subsequently expanded coverage into its current format in 2011. Thus *FBCNS* provides a unique publishing platform for all research promoted by IBBS. For the first time, researchers have a journal dedicated to publishing their work in molecular, physiological and pharmacological aspects of brain fluids and barrier systems. Many diverse neurological disorders are associated with abnormalities and/or malfunction of the brain barriers and fluid systems. Recently, the journal has published on such diverse topics as barrier function in cerebral malaria [1], multiple sclerosis [2], hypo- and hyperglycaemia [3], neuroinflammation [4], gene expression in secretory epithelia [5], intranasal drug delivery into brain [6], fetal/neonatal-onset hydrocephalus [7,8] and adult-onset hydrocephalus [9,10]. In the last few years there have been some exciting developments in research on fluid circulation into, out of, and within the brain that has been facilitated by the application of new imaging and molecular techniques e.g. [11]. The classical view of CSF circulation and flow first outlined by Cushing early in the 20^th^ century [12] has been challenged and extended and this has been highlighted in a recent review article in *FBCNS*[13]. These are exciting and controversial areas of research and *FBCNS* provides an excellent forum to publish and discuss these new ideas.

*Fluids and Barriers of the CNS* has an international Editorial Board comprising almost sixty distinguished researchers from eighteen different countries. Fourteen of these members also comprise the steering council of the *IBBS* and the journal is delighted that IBBS president Lester Drewes has recently become Co-Editor in Chief of *FBCNS*. Readers will find reciprocal links on both websites which will increase exposure and be mutually beneficial for both organisations. Society members will receive a discount on the article processing charge for papers published in *FBCNS* and will receive regular email updates from the journal. *FBCNS* offers members a route to fast publishing with full high-quality peer review.

### Open access publishing and peer review

*Fluids and Barriers of the CNS* is proud to be an *Open Access* journal: publication is online only and available to anyone in the world with internet access. This is particularly important for disseminating knowledge world-wide and ensuring rapid communication for research. Articles are free to read, to copy and distribute and the author retains the copyright. The cost of open access publishing is met by the author, their institution, or their funding source: many funding bodies insist on open access publishing and provide enabling funds. For those unable to meet the charge, the publisher and editors have access to a limited number of waivers. There are other great advantages for open access publishing: there is no page limit, no extra cost for colour, no restraints on back-up material such as raw data files, and most importantly very good exposure to the scientific community.

An essential element in the publishing process is quality peer review. For this, we rely on members of our editorial board as well as on other experts in the field. We aim to only publish papers based on sound and high quality research. A constructive and carefully-considered review almost always results in an appropriate response from the authors and an improved end product. There have been many online start-up publishers in recent years but the quality of the published product is totally dependent on a rigorous peer-review process as practised by *FBCNS.* Even with our stringent peer review, we also ensure a rapid peer review process, such that the average time from submission to a first decision is less than 4 weeks. In turn, every effort is made by the editors to clarify differences of opinion between the reviewers, and avoid multiple rounds of review where appropriate. We would like to take this opportunity to thank all those who have helped us with this critically important function in the past few years.

### Journal progress

Scientific output in the areas of blood–brain barrier and cerebrospinal fluid has increased by 100% and 60%, respectively since we started publishing in 2004. The number of publications in *FBCNS* has increased steadily with 47 articles in 2013 and the first 5 months of 2014. In 2013, we published a collection of fourteen articles on *Emerging Models and Novel Techniques for BBB and BCSFB studies* which were guest-edited by Elizabeth de Lange, Britta Engelhardt and Danica Stanimirovic (http://www.fluidsbarrierscns.com/series/brain_barriers). These have received much attention and been highly accessed by our readers, some receiving around 5000 accesses since publication last year [14-18]. This reflects the incorporation of the wider remit with the blood–brain barriers, as well as the burgeoning interest in brain barriers and fluids, with many new investigators entering this exciting and expanding field. Additionally, we have an ongoing article collection on *Hydrocephalus: Promoting Research for Improved Outcomes,* also with highly accessed papers [19,20]. In this context, the editors are actively looking for potential guest editors who would like to head up thematic issues (article collections) in expanding areas on behalf of the journal.

As an open access journal, *FBCNS* benefits from high visibility worldwide with its access and readership continuing to grow enormously in recent years; over the course of 2013, we had an average of 25,000 accesses per month, and December 2013 saw a record of 31,000 accesses. In addition to our international readership and Editorial Board, we are pleased with the level of international collaboration demonstrated across our published content. This international collaboration can be demonstrated by the proportion of articles that have been produced by researchers from several countries, which is currently 40%, as indicated in data provided by SCImago (http://www.scimagojr.com/journalsearch.php?q=19900193571&tip=sid&clean=0). Using available data, an unofficial Impact Factor of 2.95 has been calculated for the journal. Whilst this is only an estimate, this is also encouraging for a relatively new journal, and we hope for the actual Impact Factor to be announced in the near future. *Fluids and Barriers of the CNS* aims to be a first choice for investigators publishing on any topic related to the normal and pathological fluids and barriers of the CNS. The new affiliation with IBBS will help towards that end.
